# A review of the early stages and host plants of the genera *Eumerus* and *Merodon* (Diptera: Syrphidae), with new data on four species

**DOI:** 10.1371/journal.pone.0189852

**Published:** 2017-12-19

**Authors:** Antonio Ricarte, Gabriel J. Souba-Dols, Martin Hauser, Mª. Ángeles Marcos-García

**Affiliations:** 1 Centro Iberoamericano de la Biodiversidad (CIBIO), University of Alicante, San Vicente del Raspeig, Alicante, Spain; 2 Plant Pest Diagnostics Branch, California Department of Food and Agriculture, Sacramento, California, United States of America; Biocenter, Universität Würzburg, GERMANY

## Abstract

The genera *Eumerus* and *Merodon* (Diptera: Syrphidae) have a high taxonomic diversity (300+ species altogether), but life histories of most species are unknown. In addition, these hoverfly genera are recognised to be pests (ornamental plants and vegetable crops). In this paper, early stages of four hoverfly species are described, *Eumerus hungaricus* Szilády, 1940, *Eumerus nudus* Loew, 1848 and *Merodon geniculatus* Strobl, 1909, from Spain, and *Eumerus strigatus* Walker, 1859, from California, USA. Larvae of *E*. *nudus* were obtained from swollen roots of *Asphodelus cerasiferus* J. Gay. Larvae of *E*. *hungaricus* were found in bulbs of *Narcissus confusus* Pugsley. The host plant of the examined specimen of *Eumerus strigatus* is unknown. Larvae of *M*. *geniculatus* were reared from bulbs of different species of *Narcissus* L. Scanning electron microscope imaging was used to study and illustrate the anterior respiratory processes, pupal spiracles and posterior respiratory processes of the new early stages. A compilation of all available information on the early stages and host plants of *Eumerus* (21 spp.) and *Merodon* (15 spp) is provided, as well as an identification key to all known larvae/puparia of these genera. *Eumerus elavarensis* Séguy, 1961 is proposed as a new synonym of *E*. *hungaricus* and first data of this species are reported from Austria, Bulgaria, Spain and Turkey. In *Eumerus*, larvae are alleged to rely on the previous presence of decay organisms, but in the larvae of *E*. *nudus* the sclerotisation and size of the mandibular hooks suggest that this larva can generate decay from intact plant tissue.

## Introduction

As a biodiversity hotspot, and with many unique species, the Mediterranean is an area important for conservation [1]. Wild species of animals and plants have adapted their life cycles to the characteristic water deficit during summer and many plant species have developed underground storage organs (bulbs, tubers or swollen roots), for example *Amaryllidaceae*, *Xhantorrhoeaceae* (incl. *Asphodelaceae*) or *Hyacinthaceae* plants [2, 3]. These are known to the host plants of hoverfly larvae of the genera *Eumerus* Meigen, 1822 and *Merodon* Meigen, 1803 hoverflies (Diptera, Syrphidae) (e.g. [4, 5]). The genera *Eumerus* and *Merodon* have a very high species diversity within the Mediterranean region, including Europe and Turkey [6]. Adults feed on pollen and nectar and are likely to play a key role in the pollination of certain plants [4, 7, 8, 9]. In Germany, *Merodon rufus* Meigen, 1838 seems to feed specifically on *Anthericum liliago* L. and is alleged to be a specialised pollinator of this plant [10]. In Cabañeros National Park, Spain *Merodon luteihumerus* Marcos-García, Vujić and Mengual, 2007 feeds almost exclusively on pollen of *Urginea maritima* Baker [9], while their larvae feed on the bulbs of this same plant [4]. Many known larvae of *Eumerus* and all those of *Merodon* develop in underground storage organs of geophytes, feeding either in live plants (e.g. *Merodon*) or rotting parts detached from the plant (e.g. *Eumerus obliquus* (Fabricius, 1805) [4, 11, 12]).

*Eumerus* is a widely distributed genus in the Old World, especially rich in species within the Mediterranean, Central Asian and South African regions. A lesser species diversity is found in South East Asia, with the diversity steadily declining towards Australia. *Eumerus* is known as far east as New Caledonia and Fiji [13]. The New World has no native species of this genus, but several introduced species are known in North and South America [14, 15, 16, 17, 18, 19, 20, 21, 22]. *Eumerus* is one of the largest genera in the Palaearctic region with more than 160 species [23, 24, 25, 26, 27, 28].

The genus *Merodon* is native to the Palaearctic and Ethiopian regions and there are over 160 described species, of which 120 are found in Europe [6, 29, 30]. *Merodon* has more endemic species in one area of Europe than any other hoverfly genus [31]; for example, the estimated level of endemism of *Merodon* in the Iberian Peninsula is almost 50% [32]. Some regional studies have provided important information to fill in the gap in the taxonomic, distributional and phenological knowledge of *Merodon* hoverflies (e.g. [29, 32, 33, 34]) but further investigations are required to understand better the diversity and conservation of both *Eumerus* and *Merodon* [6]. Furthermore, loss of traditional agricultural practices and uses of the land negatively affects biodiversity [35] and some studies start to suggest that populations of some wild species of *Eumerus* and *Merodon* might be influenced by phenomena such as habitat encroachment or fragmentation [9, 10].

In comparison with the high species diversity of *Eumerus* and *Merodon*, little is known about their life histories. Apart from those of *Merodon equestris* (Fabricius, 1794) [36, 37], the first early stages of *Merodon* hoverflies found in the wild were those described by Ricarte et al. [4], who also provided descriptions of three species of *Eumerus* from Spain. Speight and Garrigue [12] found larvae of three species of *Eumerus* from decaying swollen roots of *Asphodelus ramosus* L. and *A*. *albus* Mill. (*Xhantorrhoeaceae*). Andrić et al. [11] described and DNA-barcoded the larva of *Merodon avidus* Rossi, 1790 found in bulbs of *Ornithogalum* L. (*Hyacinthaceae*) and in the surrounding soil. Finding early stages of these hoverfly genera in the wild has proven difficult due to their obscure breeding sites [11, 29].

Some *Eumerus* and *Merodon* species such as the large narcissus bulb fly, *M*. *equestris*, the small bulb flies, *Eumerus strigatus* (Fallén, 1817) and *Eumerus funeralis* Meigen, 1822, and the ginger maggot, *Eumerus figurans* Walker, 1859, are known to cause important damage in plants with agricultural and horticultural interest [38]. As a result, abundant literature on pest control is available (e.g. [39, 40]). In fact, countries such as Chile, Costa Rica, Mexico, Iceland or New Zealand consider these species as dangerous pests due to their dependence on storage organs of imported commercial plants such as *Narcissus*, *Hyacinthus*, *Lillium* or *Allium* [5]. Furthermore, countries such as Bermuda or Iceland consider all species of *Eumerus* and *Merodon* as harmful due to their potential as agricultural pests, even if the life cycles of most species are still unknown [5].

The general aim of the present study is to understand better the biology and functional morphology of *Eumerus* and *Merodon* larvae by studying the early stages of a *Merodon* species and three *Eumerus* species and providing data on their host plants. All available information on the early stages and host plants of these two hoverfly genera is compiled and systematically presented. In addition, an up-to-date key to the known puparia of *Eumerus* and *Merodon* is provided to facilitate the identification of larvae found both in natural and agricultural conditions.

## Materials and methods

Fieldwork to search for early stages of *Eumerus* and *Merodon* in underground storage organs of geophytes took place in different localities of Spain. In La Font Roja Natural Park, Alicante, South-Eastern Spain, *Eumerus* larvae were collected in the swollen roots of bulbs of *Asphodelus cerasiferus* J. Gay (*Xanthorrhoeaceae*) in 2009–2010. In Sierra de Béjar, Salamanca, Central-Western Spain, *Eumerus* larvae were found in bulbs of *Narcissus confusus* Pugsley (*Amayllidaceae*) in 2010. In Sierra de Mariola Natural Park, Alicante, South-Eastern Spain, larvae and puparia of *Merodon geniculatus* Strobl, 1909 were obtained from bulbs of different *Narcissus* species in 2010. In all cases, bulbs were dug out to be checked for signs of larval feeding, tunnels or decomposed tissues. Non-attacked bulbs were buried again to facilitate their regeneration. The *Narcissus* species were identified by Dr Segundo Ríos (University of Alicante). The studied puparium (+ emerged female) of *E*. *strigatus* originates from an unknown host plant in California and it was stored in the California Department of Food and Agriculture, USA (CDFA). All required permits and approvals were obtained for the field work from the authorities of the visited protected areas (La Font Roja Natural Park and Sierra de Mariola Natural Park). No protected insect species was sampled.

Larvae were transported to the laboratory and reared in plastic boxes with mesh at the top and with their original host plant. Boxes were kept in a chamber under controlled conditions, at 20°C, 65–85% humidity and without light. Boxes were inspected daily to find puparia, which were transferred individually to Petri dishes until adult emergence. When possible, the dates were recorded of the finding of a larva/puparium in the field, puparium formation and adult emergence. Emerged adults were identified using Stackelberg [41], Vujić and Šimić [42] and Speight & Garrigue [12], for *Eumerus*, and Marcos-García et al. [32] for *M*. *geniculatus*. The *E*. *strigatus* female was also confirmed genetically with DNA sequences of COI.

Larvae were described from their third larval stage, which was distinguished from other stages by having two differentiated discs on the first abdominal segment dorsally [43]. Larvae were preserved in 70% ethanol after immersion in cold water and boiling for about 4 minutes, with the purpose of fixation. For their study, puparia were cleaned with a fine paint brush after soaking in distilled water for 24h to soften materials covering the specimen; before cleaning, puparia were individualised in Eppendorf tubes with water to be treated in an ultrasonic bath at 50Hz for individual periods of 5 min, up to 25 minutes in total (individual periods of ultrasounds lasted 5 min in order to avoid pupal spiracles to be detached from the puparium). Once prepared for examination, larvae and puparia were studied with a stereo microscope.

For description of early stages, body size was measured as the length from the anterior margin of the prothorax to the anus in ventral view. Height and width of early stages were measured at their maxima. For *Eumerus*, the size of the posterior respiratory process (PRP) was measured as the distance between the transverse ridge and the centre of the spiracular plate (a) and expressed as a proportion of the width at the transverse ridge level (b). For *Merodon*, dorso-ventral height at the base of the PRP (c) was expressed as a proportion of the width at the base (d) [4]. Measurements were made with a LEICA M205C stereo microscope and the software Leica Application Suitie v.4.8. To describe the ornamentation of the anterior respiratory processes (ARP), pupal spiracles and PRP, photos were made with a HITACHI S-3000N scanning electron microscope (SEM). Head skeletons were obtained from the antero-ventral margin of emerged puparia. All puparia were soaked in a solution of KOH for 30 minutes and the head skeleton was removed with pins. Head skeletons were preserved in glycerine and studied in glycerine or 70% ethanol. Morphological terminology of early stages follows Hartley [43] and Rotheray [44], except for that of the head skeleton that follows Hartley [45], Roberts [46] and Rotheray & Gilbert [47]. Morphological terminology of adults follows Thompson [48]. Species distribution follows Speight [6] and locality data of the material examined in the present paper.

Examined material is deposited in the following collections:

CEUA, Entomological Collection of the University of Alicante, CIBIO Research Institute, Spain

CSCA, California State Collection of Arthropods, Department of Food and Agriculture, Plant Pest Diagnostic Branch, Sacramento, USA

MNHNP, Museum National d’Histoire Naturelle, Paris, France; RMNH, Naturalis (Nationaal Natuurhistorisch Museum), Leiden, The Netherlands

Abbreviations used in the text and/or figures are as follow:

ARP: anterior respiratory processes
c: spiracular scar
d: dorsal cornu
l: mandibular lobe
m: mandibular hook
o: spiracular openings
PRP: posterior respiratory process
r: transverse ridge
s: spiracular seta
t: accessory tooth
v: ventral cornu

## Results

### Descriptions of new *Eumerus* and *Merodon* early stages

#### *Eumerus hungaricus* Szilády, 1940. = *Eumerus elaverensis* Séguy, 1961 syn. nov.

New to Austria, Bulgaria, Spain and Turkey

**Puparium. Shape and dimensions:** length: mean 6.35 mm (range 6.19–6.55); width: mean 4.59mm (range 4.40–4.80); height: mean 4.08mm (range 4.03–4.11) (n = 3); sub-circular in cross-section, slightly tapered anteriorly, pale brown in colour.

**Fig 1:**
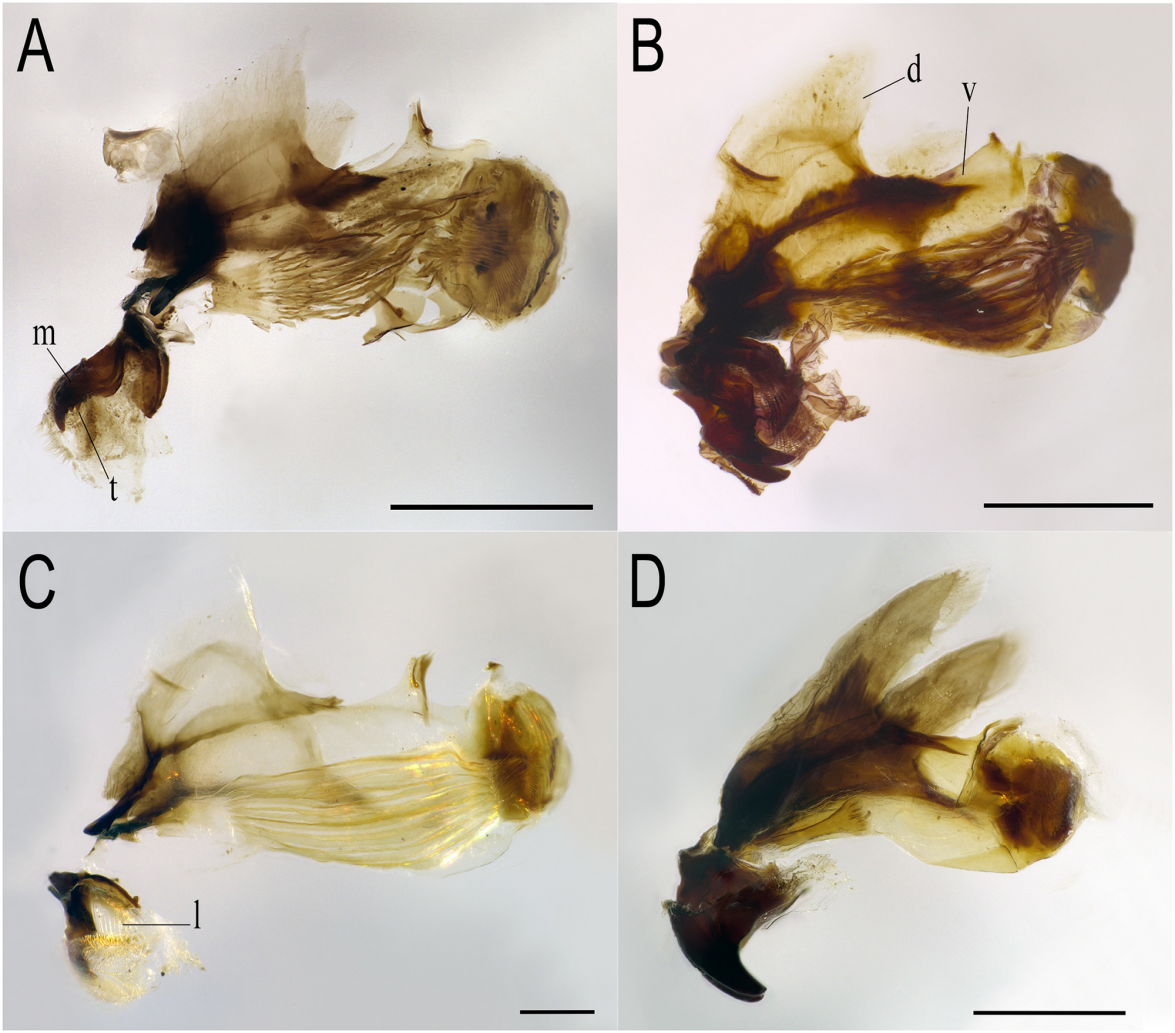
Head skeletons of *Eumerus* and *Merodon* larvae, lateral view. (A) *Eumerus hungaricus*. (B) *Eumerus nudus*. (C) *Eumerus strigatus*. (D) *Merodon geniculatus*. Abbreviations: d, dorsal cornu; l, mandibular lobe; m, mandibular hook; t, accessory tooth; v, ventral cornu. Scale lines: A, B and D = 0.5mm; C = 0.2mm.

**Head skeleton:** (Fig 1A): mandibles sclerotised, with accessory teeth present; dorsal cornu tapering posteriorly, like a shark fin, in profile view.

**Fig 2:**
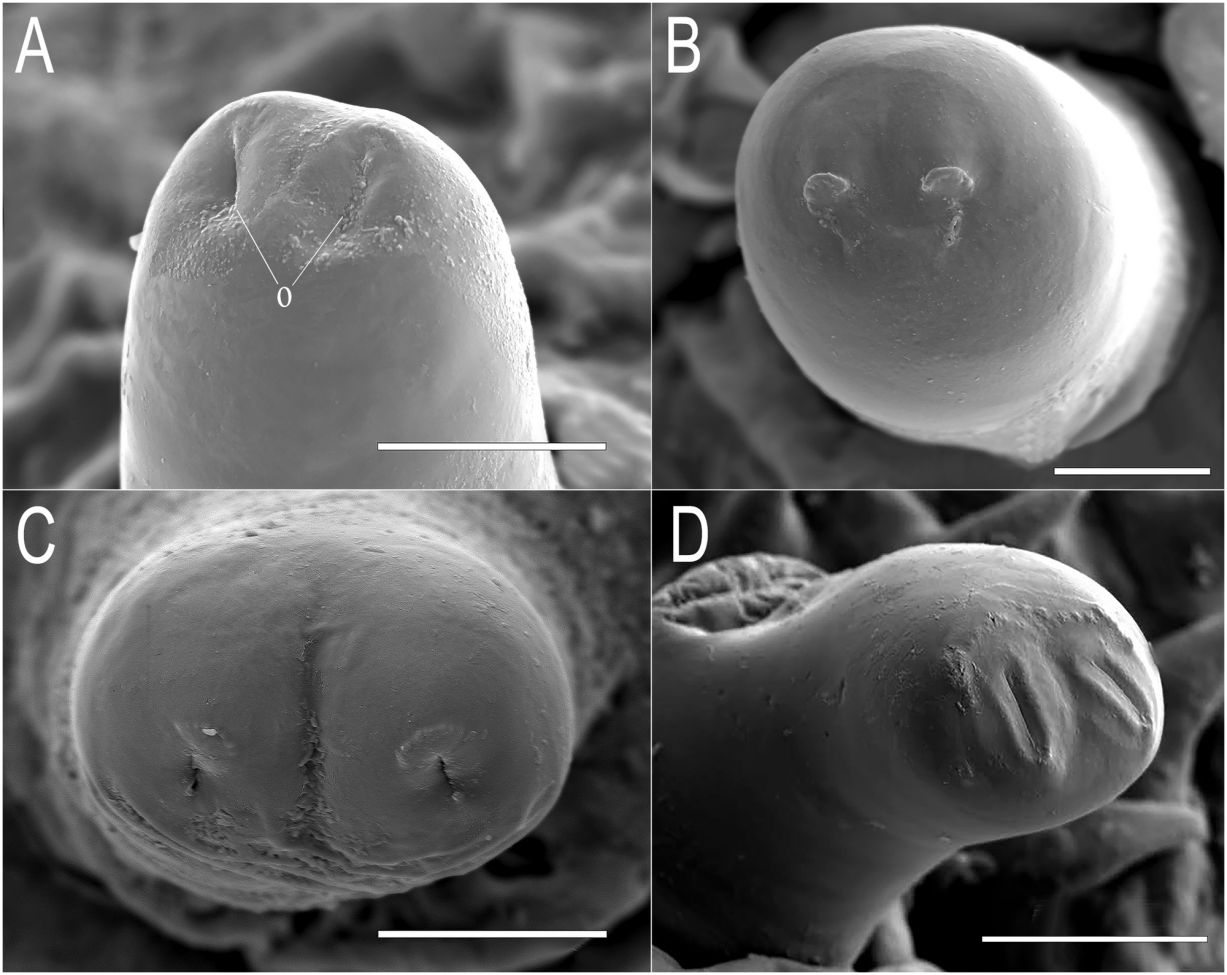
Anterior respiratory processes of *Eumerus* and *Merodon* larvae. (A) *Eumerus hungaricus*. (B) *Eumerus nudus*. (C) *Eumerus strigatus*. (D) *Merodon geniculatus*. Abbreviations: o, spiracular openings. Views: apical (B and C), apico-lateral (A and D). Scale lines: A and B = 25μm; B = 20μm; D = 50μm.

**Thorax:** ARP 0.1 mm long by 0.07 mm width, cylindrical in shape, slightly tapered towards the apex and curved to the centre of the body, light brown in colour, apex with two linear spiracular openings (Fig 2A); mesothoracic prolegs absent.

**Fig 3:**
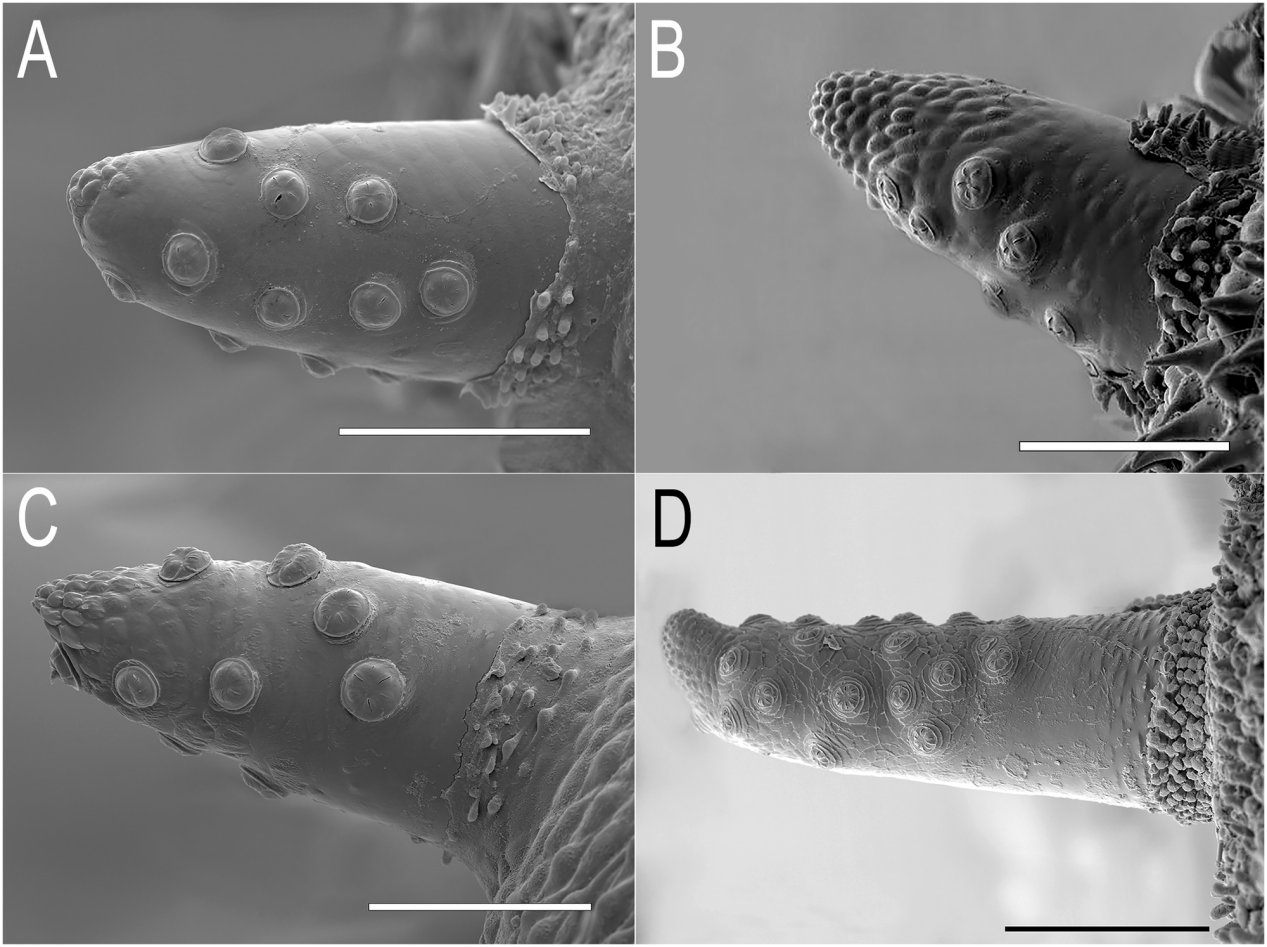
Pupal spiracles of *Eumerus* and *Merodon* puparia. (A) *Eumerus hungaricus*. (B) *Eumerus nudus*. (C) *Eumerus strigatus*. (D) *Merodon geniculatus*. Scale lines: A, B and C = 0.1mm; D = 0.2mm.

**Fig 4:**
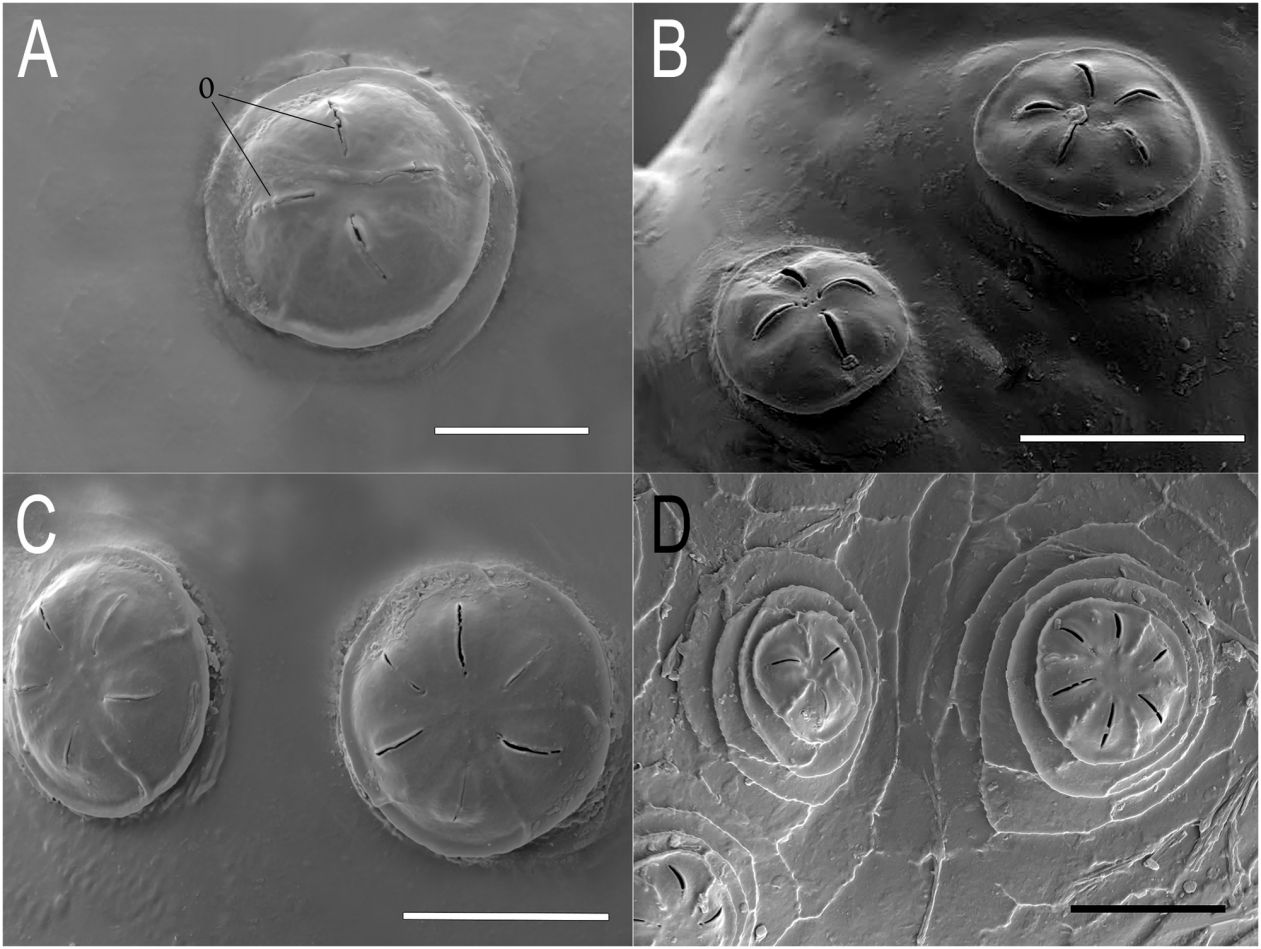
Detail of the tubercles bearing spiracular openings in the pupal spiracles of *Eumerus* and *Merodon* puparia. (A) *Eumerus hungaricus*. (B) *Eumerus nudus*. (C) *Eumerus strigatus*. (D) *Merodon geniculatus*. Abbreviations: o, spiracular openings. Scale lines: A = 10μm; B and D = 25μm; C = 20μm.

**Fig 5:**
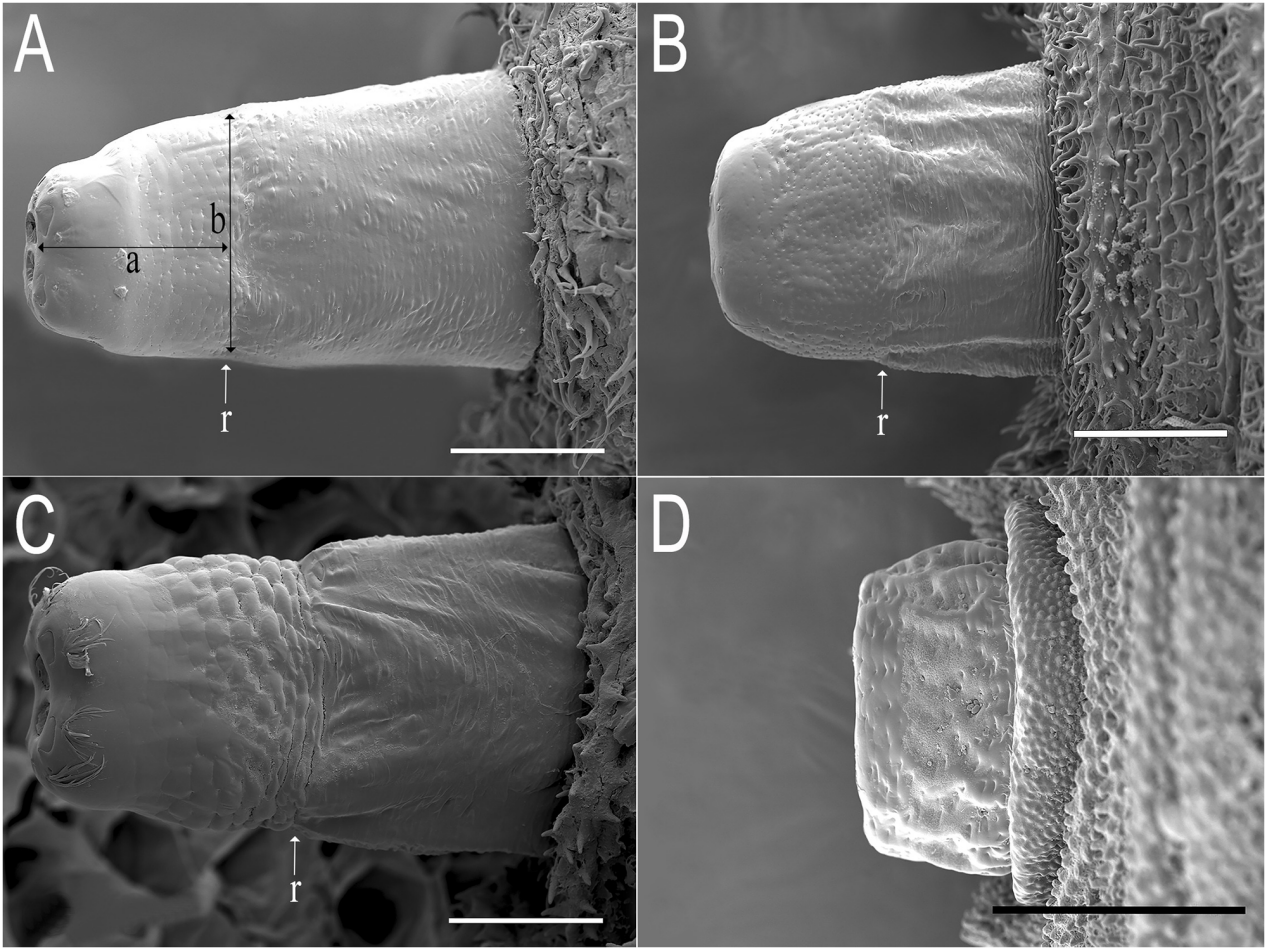
Posterior respiratory processes (PRP) of *Eumerus* and *Merodon* puparia, dorsal view. (A) *Eumerus hungaricus*. (B) *Eumerus nudus*. (C) *Eumerus strigatus*. (D) *Merodon geniculatus*. Abbreviations: a, distance between the transverse ridge and the centre of the spiracular plate; b, width at the transverse ridge level; r, transverse ridge. Scale lines: A and C = 0.2mm; B = 0.25mm; D = 0.5mm.

**Fig 6:**
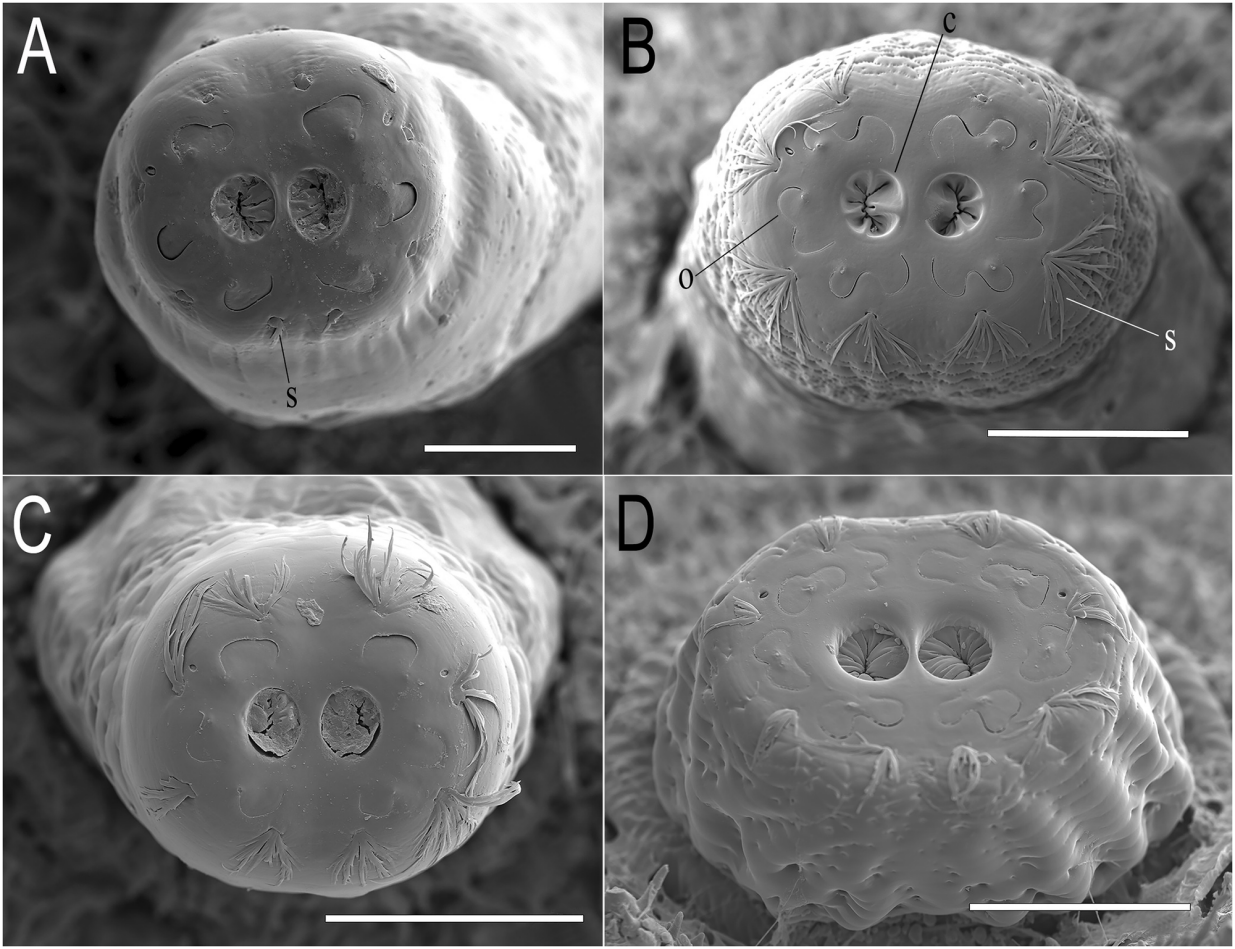
Posterior respiratory processes (PRP) of *Eumerus* and *Merodon* puparia showing the spiracular plate. (A) *Eumerus hungaricus* (only the basis of the spiracular setae are left in this specimen). (B) *Eumerus nudus*. (C) *Eumerus strigatus*. (D) *Merodon geniculatus*. Abbreviations: c, spiracular scar; o, spiracular openings; s, spiracular seta. Views: apical (A, B and C), apico-ventral (D). Scale lines: A = 0.1mm; B and C = 0.2mm; D = 0.25mm.

**Abdomen:** first abdominal segment with pupal spiracles 0.29 mm long, separated by 6× their length, bearing on the dorsal surface irregularly-spaced, round-shaped tubercles (Fig 3A); each tubercle with 4–5 linear spiracular openings, arranged radially (Fig 4A); pupal spiracle surface shiny and almost smooth with irregular fine and shallow marks, granulated at the apex (Fig 3A); prolegs present bearing groups of small hooks lacking conspicuous planta; anal segment elongate, bearing three pairs of lappets, the first pair virtually absent, the second inconspicuous, divided into two projections, and the third well developed; PRP: inclined upward from the transverse ridge to the apex; transverse ridge conspicuous; a = mean 0.41mm (range 0.37–0.43); b = mean 0.43mm (range 0.41–0.43); a/b = 0.95 (n = 3); PRP shiny and brown in colour, transverse ridge conspicuous; PRP with fine transversal marks below the ridge and shallow punctures above; the final part of the PRP, after the punctured area, is smooth and curved, smaller in diameter than the rest of the PRP; PRP asymmetric, especially in the section from the transverse ridge to the PRP apex (Fig 5A); spiracular plates with three pairs of curved spiracular openings, with four pairs of setae around the margin of the plate (in the examined specimens, all setae were broken) (Fig 6A).

**Species distribution:** Austria, Bulgaria, Iberian Peninsula (Gibraltar; Alicante Salamanca and Valencia provinces, Spain), France, Hungary, Romania, Switzerland, Italy, Macedonia, Turkey.

**Taxonomic notes:** *E*. *hungaricus* was described from males collected in central Hungary [49]. The type material of *E*. *hungaricus* is destroyed [50], but Szilády illustrated in the original description the metaleg of his new species. The metaleg of *E*. *hungaricus* is rather characteristic within the genus; the femur is swollen and has long pile ventrally (length of longest pile same as maximum width of femur) and the posterior side of tibia has a conspicuous bump bearing pile. The lateral margins of tergites 3^rd^ and 4^th^ are adorned with conspicuous long setae, similar to *Eumerus pulchellus*. Doesburg [50] redescribed the male of *E*. *hungaricus* and described the female for the first time. Doesburg [50] designated a neotype (as ‘neo-holotype’) from a series of 57 males and 14 females (all from Castiglione dei Pepoli, Bologna, Italy 16/26.VII.1957, V. van der Goot leg). According to the International Code of Zoological Nomenclature, Article 75.3.7 [51], a neotype designation is only valid when “*a statement that the neotype is*, *or immediately upon publication has become*, *the property of a recognized scientific or educational institution*, *cited by name*, *that maintains a research collection*, *with proper facilities for preserving name-bearing types*, *and that makes them accessible for study”* but such a statement has never been published. In fact, Doesburg’s neotype was deposited in his own collection. Furthermore, it is questionable if a neotype was needed for the “*purpose of clarifying the taxonomic status”* (Article 75.3.1.), because the identity of the species was never in doubt (despite the type specimen being destroyed), because of the detailed description and the drawings. *E*. *hungaricus* is just a rarely collected species and Séguy likely did not check all the west Palaearctic *Eumerus* descriptions when describing *E*. *elaverensis* in 1961, and therefore overlooked *E*. *hungaricus*. Despite us not agreeing with the validity of Doesburg’s neotype designation, we agree that the specimens Doesburg [50] studied are conspecific with Szilády’s *E*. *hungaricus*. One of us (Martin Hauser) examined a specimen of *E*. *hungaricus* from Doesburg’s series (labelled as Neo-paratype), as well as three (2 males and 1 female) syntypes of *E*. *elaverensis*, which was described from France [52]. The examined neo-paratype of *E*. *hungaricus* and the two male syntypes of *E*. *elaverensis* shared the metatibia morphology, the long hairs at the lateral margins of abdomen (this character is shared by a very small group of *Eumerus* species) and the yellow apex of the 4^th^ tergite. For these and other morphological similarities, we consider all these specimens to be conspecific and we propose *E*. *elaverensis* as a junior synonym of *E*. *hungaricus*.

**Examined material:** AUSTRIA: 1 male, Krems-Land Dist. S-Hänge, NE Dürnstein, 410m, 49°23’58”N 15°31’31”E, 2.VIII.1995 [Coll. M. Hauser]. BULGARIA: 1 male, collected 11-6/25-6-1998 by P.V. Attanassova [Coll. J. Smit]. FRANCE: 1 male, Corse, Asco, 620 m, 5.VII.1967, leg. V.S. van der Goot (det. van der Goot); 1 male, Provonce Mtgn de Lure, Lauzon-Tal b. Montlaux, 500m, 12.VIII.1985, leg W.Schacht [RMNH]; 1 male, 1 female, Hautes-Alpes, Aileroide camping, 44°52’40”N, 6°26’00”E, 22.VII.2010, 1600m.a.s.l. J. van Steenis. SPAIN: 2 females, Alcoy, Alicante, 1100-1250m, Sierra de la Font Roja, Sierra de Menechaor, Mediterranean oak forest, 38°40’N 0°32W, 15.VI.2003, J. van Steenis & M.P. van Zuijen; 2 males, Valencia, Ontinyent, Sierra de Mariola, pas on 950m, 38°43’N 0°32’W, on ground, 18.VI.2003, J. van Steenis, B. Wakkie & M.P. van Zuijen; 3 adults with puparia (2 males and 1 female; emergence: 2/5/2010) reared from larvae collected in live *Narcissus confusus* bulbs at Los Canalizos (1024m), Sierra de Béjar, Salamanca, on 21/3/2010, by M.A. Marcos-García [CEUA]. TURKEY: 1 male and 2 females, Afyon, Sultandaglari, 10 km S of Cay, 1200 m, 8.VIII.1981, leg. H. Coene, J. Lucas & B. van Oorschot (det. J. Lucas); 1 female, Afyon, Sultandaglari, 15 km S of Cay, 1400 m, 1.VIII.1981, leg. H. Coene, J. Lucas & B. van Oorschot (det. J. Lucas) [RMNH]. Type material examined: *E*. *elaverensis*: 1 male, Lardy, 5.VI.37 (handwritten) / g. Portevin 1937 (handwritten) / syntypus ♂ (handwritten in red) / *Eumerus elaverensis* d. Hauser 05 (handwritten in red). 1 male, Broût-Vernet, H. du Buysson / Museum Paris / *elaverensis* syntypus ♂ (handwritten) / syntypus ♂ (handwritten in red). 1 female, Broût-Vernet, H. du Buysson / Museum Paris / syntypus ♀ *elaverensis* (handwritten in red) [MNHNP]. *E*. *hungaricus*: 1male, Castiglione dei Pepoli, Bologna, Italia, 600-800m, 17.VII.1957, v. d. Goot Theowald / *Eumerus hungaricus* Szil. ♂ (hand written), Neo-paratype (hand written in red), det. V. Doesburg [CSCA].

#### *Eumerus nudus* Loew, 1848

**Third instar (L3) larva. Shape and dimensions:** length: mean 14.93mm (range 12.08–17.77); width: 5.41mm (5.01–5.68); height: 5.31mm (4.86–5.65) (n = 4); larvae sub-cylindrical in cross-section, tapering anteriorly, anal segment elongate.

**Head:** antennae and maxillary organs on a pair of rounded to oval bulbous projections with a mid-surrounding dark band on the sides; dorsal lips bearing small dark setae arranged in numerous rows (7–9) and mandibular lobe coated with setae as well.

**Head skeleton:** (Fig 1B): mandibular hooks sclerotised and mandibular lobes fleshy and fused with the mandibles, with dorsal cornu tapering towards the apex, fin shaped, in profile view; mandibular hooks 0.6mm long, with accessory teeth and, in apical view, separated at apex by the same distance than basal width.

**Thorax:** ARP prominent, 0.13 mm long, 0.07 mm wide, almost cylindrical in shape, light brown in colour, with two spiracular openings across the dome apex (Fig 2B); mesothoracic prolegs absent.

**Abdomen:** integument spiculated, without setae; sensilla with at least two setae each; prolegs on abdominal segments 1–6 bearing two parallel rows of crochets (anterior row with 4–5 crochets; posterior row with 2–4 smaller crochets); anal segment elongate (2.20 mm long, about 1.2× longer than the 6^th^ abdominal segment), with ventral part longer than dorsal and then segment oriented upward in appearance; three pairs of conspicuous lappets; middle lappets divided into two separate conic projections, 1^st^ and 2^nd^ conic, 3^rd^ elongated, longer than the other pairs of lappets; PRP: a = 0.46mm (0.39–0.56); b = 0.65mm (0.59–0.72); a/b = 0.74 (n = 4); dark brown, shiny, sub-elliptical in cross section; below transverse ridge strongly striated longitudinally, finely transversally, above coriaceous and smoother towards the apex (Fig 5B); spiracular plate with three pairs of *ω*-shaped spiracular openings and four pairs of multibranched setae around the margin of the plate. Spiracular scars in a pair of rounded depressions (Fig 6B).

**Puparium. Shape and dimensions:** length: mean 9.08mm (range 8.02–9.70); width: mean 5.65mm (range 4.86–6.22); height: mean 5.09mm (range 4.00–5.56) (n = 8); light brown in colour.

**Head skeleton:** see under larva description (Fig 1B).

**Thorax:** ARP 0.11mm long by 0.07mm width, cylindrical in shape, slightly swollen to the apex, yellowish to dark brown in colour, apex with two openings (Fig 2B); mesothoracic prolegs absent.

**Abdomen:** first abdominal segment with pupal spiracles 0.3mm long, separated almost 8× their length, surface smooth at the base and densely granulated towards the apex, bearing irregularly-spaced, round-shaped tubercles (Fig 3B); each tubercle with 3–5 spiracular openings, arranged radially (Fig 4B); pupal spiracle surface shiny and smooth at the base and coriaceous between tubercles towards the apex (Fig 3B); PRP: a = 0.39mm (range 0.21–0.44); b = 0.61mm (range 0.41–0.72); a/b = 0.64 (n = 8); surface below ridge irregularly wrinkled longitudinally and diagonally, finely striated transversally; above transverse ridge, punctured and smoother to the apex (Fig 5B); spiracular openings sinuous in shape (Fig 6B).

**Species distribution:** from Spain to the former Yugoslavia and Turkey, through Southern France and Italy (also in Sicily); Romania; Northern Africa: Morocco, Algeria and Tunisia.

**Examined material:** 4 larvae obtained from swollen roots of *A*. *cerasiferus* at the Menetjador peak (P.N. Font Roja, Alcoy, Spain) by M.A. Marcos-García and A. Ricarte which were preserved in ethanol; Puparia: 4 larvae obtained from swollen roots of *A*. *cerasiferus* at the Menetjador peak (P.N. Font Roja, Alcoy, Spain) by J. Quinto from which 2 males and 2 females emerged on 3/6/2009; 4 larvae obtained from *A*. *cerasiferus* at the Menetjador peak (P.N. Font Roja, Alcoy, Spain) by M.A. Marcos-García and A. Ricarte from which 1 male and 3 females emerged between 26/4/2010 and 2/5/2010 [CEUA].

#### *Eumerus strigatus* (Fallén, 1817)

**Puparium. Shape and dimensions:** length: 5.76mm; width: 4.29mm; height 3.32mm (n = 1); sub-cylindrical, brown in colour.

**Head skeleton:** (Fig 1C): mandibular hooks sclerotised, not massive, with accessory teeth present; dorsal cornu tapering posteriorly in profile view, little sclerotised, almost entirely translucent; lips coated in setae and mandibular lobes with conspicuous ridges present after removal of the head skeleton from the puparium.

**Thorax:** ARP 0.1mm long, width of 0.72mm and height of 0.04mm, oval in shape and light brown in colour, apex with a groove separating 2 linear spiracular openings (Fig 2C); mesothoracic prolegs absent.

**Abdomen:** integument smooth, bearing transversal rows of small hooks along the body; first abdominal segment with 0.34mm long pupal spiracles, separated by 4.5× their length, bearing irregularly-spaced, round-shaped tubercles (Fig 3C); each tubercle with 5–6 spiracular openings, arranged radially (Fig 4C); pupal spiracle surface between tubercles almost smooth but granulated at the apex (Fig 3C); prolegs present on the first six abdominal segments bearing two rows of crochets; anal segment elongate, with three pairs of lappets bearing sensilla, middle ones divided into two smaller projections than those of the other pairs; PRP: a = 0.4mm; b = 0.47mm; a/b = 0.85 (n = 1); below ridge, fine transversal striations with some diagonal wrinkles; immediately above ridge with bulges, diminishing towards the apex until smooth (Fig 5C); spiracular openings U-shaped, with 4 pairs of linear and divided setae around the margin of the spiracular plate (Fig 6C).

**Distribution**: Fennoscandia south to Iberia and the Mediterranean; much of Europe through into Turkey and Russia; from the Urals to the Pacific coast (Sakhalin); Japan; introduced to North America and recorded from both Canada and the USA; introduced also to both Australia and New Zealand.

Examined material: a puparium (+ emerged adult female) obtained from a larva found in an unknown host plant in California (USA) [CSCA].

#### *Merodon geniculatus* Strobl, 1909

**Puparium. Shape and dimensions:** length: 10.14mm (9.11–12.23); width: 6.48mm (6.12–7.28); height: 5.93mm (5.56–6.88) (n = 4); sub-cylindrical in cross section; anterior extreme rounded, inclined posteriorly and flattened ventrally; pale brown in colour.

**Head skeleton:** (Fig 1D): mandibular hooks heavily sclerotised with both dorsal and ventral cornua bar-shaped in profile view; mandibular hooks 0.60mm long without accessory teeth.

**Thorax:** ARP sclerotised, 0.19mm long by 0.07 mm wide, cylindrical in shape, blackish-brown in colour, with two linear spiracular openings at the apex (Fig 2D); pupal spiracles 0.77mm long, separated by a distance of 3× their length; surface extensively reticulated with lines drawing cells that encircle the spiracular tubercles, smoother near the base and granulated towards the apex (Fig 3D); each spiracle bearing numerous domed tubercles irregularly distributed but less dense on the margin facing the centre of the segment; each tubercle with 4–5 radially arranged spiracular openings; mesothoracic prolegs absent.

**Abdomen:** integument covered with granules, with sensilla bearing a seta; anal segment retracted obliquely, with three pairs of lappets; all lappets with basal projection barely produced; middle lappets consisting of two separate projections; PRP: c = 0.83mm (0.69–0.96); d = 0.93mm (0.89–1.03); c/d = 0.89 (n = 5); black, shiny, transverse ridge not visible, entirely coriaceous, sub-elliptical in cross section (Fig 5D); spiracular plate with 4 pairs of irregularly curved spiracular openings; margin with four pairs of feathery setae; spiracular scars in a pair of two abrupt cavities in the middle of the spiracular plate (Fig 6D).

**Species distribution:** Southern France and the Iberian Peninsula, Italy, Southern parts of the former Yugoslavian countries, from Bulgaria to Greece and Turkey; North Africa (Algeria and Morocco); Mediterranean islands: Balearic Islands, Corsica, Sardinia and Malta.

**Examined material:** 1 larva obtained next from a *Narcissus dubius* bulb in El Preventori (P.N. Sierra Mariola, Alcoy, Spain) by M.A. Marcos-García from which 1 female emerged on 3/5/2010; 2 puparia obtained from *N*. *triandrus* subsp. *pallidulus* bulbs at the Botanical Garden of Torretes (Ibi, Spain) by M.A. Marcos-García and S. Ríos from which 2 females emerged in 2010; 3 puparia obtained from bulbs of *N*. *rupicola* bulbs at the Botanical Garden of Torretes (Ibi, Spain) by M.A. Marcos-García and S. Ríos from which 1 male and 2 females emerged in 2010; 1 puparium obtained from a bulb of *N*. *tazetta* at the Botanical Garden of Torretes (Ibi, Spain) by M.A. Marcos-García and S. Ríos from which 1 male emerged in 2010) [CEUA].

### Key to early stages of *Eumerus* and *Merodon* species (third stage larvae and puparia)

A key to all known larvae/puparia of *Eumerus* and *Merodon* species is provided to facilitate the identification of these genera based on early stages found both in natural and cultured situations. Keys were elaborated by examination of actual specimens and descriptions/diagnoses/illustrations published in the references provided in Table 1. Authors had not access to early stages of the species marked with an asterisk (*) in the keys.

**Table 1:** Food plants and early stages of the world *Eumerus* and *Merodon* species.

Species	Host plants^a^	Morphology^b^
***Eumerus* Meigen, 1822**		
*E*. *alpinus* Rondani, 1857	Reared from swollen roots of *Asphodelus ramosus* L. and *Asphodelus albus* Mill. (as *E*. *olivaceus* in Speight & Garrigue [12])	Puparium briefly described (as *E*. *olivaceus* in Speight & Garrigue [12])
*E*. *amoenus* Loew, 1848	Reared from *Allium* (*Alliaceae*), potato tubers, water melon, grapes, rotten paw-paw and damaged rhizomes of *Iris germanica* L. (*Iridaceae*) [57]; injurious to onion [58]	Undescribed
*E*. *barbarus* (Coquebert, 1804)	Reared from cultivated *Allium* sp. (*Alliaceae*) [6]	Undescribed
*E*. *compertus* Villeneuve, 1924	Reared from bulbs of *Cistanche phelypaea* (L.) Cout. (as *Cistanche tinctoria* (Forssk.) Beck in Waitzbauer [59]) (*Orobanchaceae*)	Larva, puparium and head skeleton described and illustrated [59]; larval characters provided in a matrix [60]
*E*. *etnensis* Goot, 1964	Reared from *Opuntia maxima* Mill. platyclades (*Cactaceae*) (as *Eumerus purpurariae* in Pérez-Bañón & Marcos-García [38])	Egg, larva, puparium and head skeleton described and illustrated (as *Eumerus purpurariae* in Pérez-Bañón & Marcos-García [38])
*E*. *figurans* Walker, 1859	Larva causes ‘considerable damage’ to lily bulbs, especially *Narcissus*, and ginger (*Zingiber* sp, Zingiberaceae) (as *Eumerus marginatus* in Hardy [61]), causing pests in ginger root cultures (Miyasaka et al. [62]); feeding in rotten corms of taro *Colocasia esculenta* (L.) Schott (Araceae) (Miyasaka et al. [62])	Undescribed
*E*. *funeralis* Meigen, 1822	Various plant genera in cultured situations (see under *E*. *tuberculatus*)	Larva and puparium described (see under *E*. *tuberculatus*)
*E*. *hispidus* Smit, Franquinho-Aguiar & Wakeham-Dawson, 2004	Adults very often found feeding in *Euphorbia* (*Euphorbiaceae*) flowers in large number (pers. comm. of J.T.Smit to M.C.D. Speight), so larvae are likely to feed on underground parts of *Euphorbia* [6]	Undescribed
*E*. *hungaricus* Szilády, 1940	Reared from bulbs of *Narcissus confusus* Pugsley (present study)	Puparium described (present study)
*E*. *latitarsis* Macquart in Webb & Berthelot, 1839	Larva occurs in decaying parts of stems of *Euphorbia canariensis* L. (*Euphorbiaceae*) [63]	Undescribed
*E*. *narcissi* Smith, 1928	Reared from cultivated *Narcissus*, probably *N*. *tazetta* L. [64]; found in a greenhouse with cultivated narcissus, ‘very likely brought from the West Coast [of USA] with bulb material’ [65]; reared from decayed narcissus [65, 66]	Undescribed
*E*. *nudus* Loew, 1848	Reared from swollen roots of *Asphodelus ramosus* L. [12] and *Asphodelus cerasifeus* J. Gay (present study)	Puparium briefly described [12]; larva and puparium described in detail (present study)
*E*. *obliquus* (Fabricius, 1805)	Reared from many decaying plants including cuttings of poinsettia (*Euphorbiaceae*) in water, fruits and vegetables [67]; reared from fruits and platyclades of *Opuntia maxima* Mill. [4]	General description of the larva [67]; detailed description and illustrations of the puparium and head skeleton [4]
*E*. *pulchellus* Loew, 1848	Reared from bulbs of *Drimia maritima* (L.) Stearn, and from swollen roots of *Asphodelus aestivus* Brot. [4]	Puparium and head skeleton described and illustrated [4]
*E*. *pusillus* Loew, 1848	Reared from bulbs of *Drimia maritima* (L.) Stearn [4]	Puparium and head skeleton described and illustrated [4]
*E*. *ruficornis* Meigen, 1822	*Scorzonera humilis* L. (*Asteraceae*) supposed to be a host plant [68]	Undescribed
*E*. *sabulonum* (Fallén, 1817)	Oviposition and first instar larvae observed on leaves of *Jasione montana* L. (*Campanulaceae*), although these larvae not confirmed to belong to this *Eumerus* species [69]	Undescribed
*E*. *sogdianus* (Stackelberg, 1952)	Reared from *Allium*, carrots and potatoes [70]	Undescribed
*E*. *speculifer* Sharp, 1899	Reared from narcissus (as *Eumerus peltatus* in Neboiss [71])	Undescribed
*E*. *strigatus* (Fallén, 1817)	Reared from onion [72], *Narcissus* (*Amaryllidaceae*) and related plants in commercial situations [54, 73, 74]; indicated as potential pest of narcissus and onion by Broadbent [73]; larva found in hyacinth bulbs and a scattered record in tulip bulbs [74]; reared from grapefruit, tomatoes and carrots [75, 76]; found in ‘dump heaps’ of ‘decayed and undesired bulb materials’ [65]; attacking iris, parsnip [55] and potatoes [77, 78]; reared from decayed narcissus (Blanton [66], by citation of Blanton & Spruijt [65]); reared from decomposed oatmeal [79]; larvae infesting onion and garlic [20, 55, 74]; larvae living inside *Fritillaria* spp bulbs (*Liliaceae*) [80]	Larva and puparium (‘pupe’) illustrated and briefly described (as *Eumerus aeneus*), and larva compared with that of *Cheilosia scutellata* [72]; life cycle described and early stage morphology briefly featured by Broadbent [73]; egg, larva and puparium described and illustrated, life history also described [74]; larva described [81] and compared with that of *E*. *tuberculatus* [55]; larva and puparium described and illustrated, table of characters to separate them from those of *E*. *tuberculatus* provided [82]; larva described and included in a key to the larvae of some British hoverfly species [83]; PRP illustrated in polar view [84]; larva and puparium briefly described and included in a key to be separated from *E*. *tuberculatus*, with pupal spiracle illustrated [85]; puparium described in detail (present study)
*E*. *tricolor* (Fabricius, 1798)	Reared from *Tragopogon porrifolius* L (*Asteraceae*) in commercial situations [86] and *Tragopogon pratensis* L. in the wild [87]	Egg, larva, puparium and head skeleton described and illustrated; life cycle described [86]
*E*. *tuberculatus* Rondani, 1857 (valid name: *E*. *funeralis*)	Reared from *Narcissus* and related plants in commercial situations [54, 71, 88]; found in ‘dump heaps’ of ‘decayed and undesired bulb materials’ [65]; reared from decayed narcissus (Blanton [66], by citation of Blanton & Spruijt [65]); commercial *Hyacinthus*, *Lilium*, *Tulipa*, *Amaryllis*, *Allium*, *Hippeastrum advenum* Herb., *Iris reginae* Horvat & M.D.Horvat [5]	Larva described and compared with those of *Syritta pipiens* [56] and *E*. *strigatus* [55]; larva and puparium described and illustrated, table of characters to separate them from those of *E*. *strigatus* [82]; larva described and included in a key to the larvae of some hoverfly species found in Britain [83]; larva and puparium briefly described and included in a key to be separated from *E*. *strigatus* [85]; head skeleton illustrated [47, 56]; larval characters provided in a matrix [60]
***Merodon* Meigen, 1803**		
*M*. *alexandri* Popov, 2010	Under laboratory conditions, first instar larvae feed on bulbs of *Scilla siberica* Haw. and *Leopoldia comosa* (L.) Parl. (*Hyacinthaceae*), although the later species does not grow in the distribution area of *M*. *alexandri*, which might be then oligophagous [89]	Undescribed
*M*. *armipes* Rondani, 1843	Apparently associated to *Muscari* (pers. comm. of D. Doczkal to M.C.D. Speight) and *Ornithogalum* [6]	Undescribed
*M*. *avidus* Rossi, 1790	Oviposition observed in *Muscari* sp. (*Hyacinthaceae*) [90]; larvae obtained from bulbs of *Ornithogalum umbellatum* L. [11]	2^nd^ instar larva described and illustrated [11]
*M*. *bombiformis* Hull, 1944	Reared from *Gladiolus* sp. in a city [91]	Larva and puparium described and illustrated [91]; comparison table of larval characters for this and other *Merodon* species [11]
*M*. *cinereus* (Fabricius, 1794)	Probably associated to spring-flowering *Crocus* L. [6]	Undescribed
*M*. *dobrogensis* Bradescu, 1982	Probably associated to *Prospero autumnale* (L.) Speta (formerly *Scilla autumnalis* L., *Asparagaceae*) [6]	Undescribed
*M*. *eques* (Fabricius, 1805)	Reared from bulbs of *Narcissus* sp. [92]	Undescribed
*M*. *equestris* (Fabricius, 1794)	Reared from *Iris*, *Narcissus* and related plants in commercial situations [36, 66, 93, 94]; known to attack *Cyrtanthus elatus* (Jacq.) Traub (as *Vallota purpurea* in Jack [36] and Douchette [37]); infestation in *Hippeastrum* hybrids (*Amaryllidaceae*) [36, 37]; unusual rearing record from *Hippeastrum* sp. [95]; reported from daffodil bulbs (*Narcissus*) intercepted in Hawaii but ‘not known to be established’ on these islands [61]; commercial *Amaryllis* [93], *Galanthus*, *Galtonia*, *Hyacinthus*, *Lilium*, *Scilla*, *Tulipa* and *Musa* [5]; bulbs of *Lycoris squamigera*; list of plants found to be infested in the United States Department of Agriculture plus general list of host plants provided by Douchette [37]	General description of larvae and puparium, as well as life cycle [36]; general description of larva and puparium (‘pupe’) [93]; egg, larva and puparium briefly described and illustrated, life history also described [96]; egg, larva (all instars), puparium and life cycle described [94]; larva and puparium described and illustrated, with PRP illustrated erroneously with three pairs of spiracular openings [81]; egg, L1, L2, L3, puparium (‘pupa’) and life history described [37]; larva described and included in a key to the larvae of some hoverfly species found in Britain [83]; PRP illustrated in polar view in Dušek & Láska [84] as *Lampetia equestris* (illustrated erroneously with three pairs of spiracular openings); larva and puparium briefly described, with PRP in dorsal (?) and polar views, pupal spiracle and puparium illustrated [85]; head skeleton described [43]; larval characters provided in a matrix [60]; comparison table of larval characters for this and other *Merodon* species [11]
*M*. *flavus* Sack, 1913	Probably associated to *Narcissus* L. [6]	Undescribed
*M*. *geniculatus* Strobl, 1909	Reared from different species of *Narcissus* L (present study)	Puparium described (present study)
*M*. *hurkmansi* Marcos-García, Vujić & Mengual, 2007	Reared from commercially grown bulbs of *Muscari comosum* (L.) Mill. (as *Merodon constans* in Ricarte et al. [4])	3^rd^ instar larva and head skeleton described and illustrated (as *Merodon constans* in Ricarte et al. [4]); comparison table of larval characters for this and other *Merodon* species [11]
*M*. *loewi* van der Goot, 1964	Probably associated to *Ornithogalum* L. (*Asparagaceae*) [97]	Undescribed
*M*. *luteihumerus* Marcos-García, Vujić & Mengual, 2007	Reared from bulbs of *Drimia maritima* (L.) Stearn [4]	Egg, 1^st^ and 3^rd^ instar larvae, puparium and head skeleton described [4]; comparison table of larval characters for this and other *Merodon* species [11]
*M*. *nigritarsis* Rondani, 1845	Reared from *Hyacinthaella pallasiana* (Ster.) Losinsk. (*Hyacinthaceae*) [98]; *Muscari racemosum* Mill. might be another host plant [6]	Undescribed
*M*. *rufus* Meigen, 1838	Probably associated to *Anthericum* L. [6]	Undescribed

^a^This column includes information on the actual and/or potential host plants of each species larva

^b^This column includes information on the availability of early stage descriptions, diagnoses, comparisons with other species and illustrations (in the original references, illustrations might represent eggs/larvae/puparia or parts of them).

-Mandibular lobes fleshy (Fig 1C) or sclerotised, anal segment elongated to contracted, but PRP always with three spiracular openings (Fig 6A and 6B) ***Eumerus***

-Mandibular lobes sclerotised and fused with the mandibles (Fig 1D), anal segment retracted obliquely and PRP with four spiracular openings (Fig 6D) ***Merodon***

### *Eumerus* species

1a. Mesothoracic prolegs present … 2

1b. Mesothoracic prolegs absent … 3

2a. Head skeleton: mandibular hook serrated apically; dorsal cornu rounded. Pupal spiracles bearing tubercles with 6–10 spiracular openings. PRP with inconspicuous vestiture … ***E*. *etnensis***

2b. Head skeleton: mandibular hook not serrated; dorsal cornu pointed. Pupal spiracles bearing tubercles with 5–7 spiracular openings. PRP with conspicuous vestiture … ***E*. *obliquus***

3a. Anal segment contracted, as long as broad or broader; only first pair of lappets well developed; PRP short, barely visible or not visible with larva/puparium in lateral view … 4

3b. Anal segment elongated to varying degrees; all three pairs of lappets developed or third pair more developed than the other pairs; PRP long, clearly visible with larva/puparium in lateral view … 5

4a. Tentorial arm heavily sclerotised; dorsal cornu shorter than ventral cornu (host plants: *Tragopogon* spp) … *E*. tricolor*

4b. Tentorial arm slightly sclerotised; dorsal cornu longer than ventral cornu (host plant: *Cistanche phelypaea*) … *E*. compertus*

5a. PRP conspicuously asymmetric above transverse ridge, specially near the apex (Fig 5A) … ***E*. *hungaricus***

5b. PRP symmetrical … 6

6a. Mandibular hook with a single accessory tooth; PRP with spicules basally … ***E*. *pulchellus***

6b. Mandibular hook with more than one accessory tooth. PRP without spicules … 7

7a. Head skeleton with large mandibular hooks, apically curved for about half of their total length (Fig 1B); PRP with spiracular openings very sinuous, clearly ω-shaped (Fig 6B) … ***E*. *nudus***

7b. Head skeleton with smaller mandibular hooks, bar-shaped, apically curved for less than half of their total length; PRP with spiracular openings U-shaped or slightly sinuous … 8

8a. Mandibular hook with four accessory teeth; PRP below transverse ridge with conspicuous transverse striations all over … ***E*. *pusillus***

8b. Mandibular hook with more than 4 accessory teeth; PRP below transverse ridge with different vestiture … 9

9a. Antenno-maxilary organs of larva separated by an inconspicuous groove or without groove; mandibular hook usually with seven accessory teeth, rarely six; PRP below transverse ridge with coarse diagonal wrinkles, faintly striated transversally (Fig 5C) … ***E*. *strigatus***

9b. Antenno-maxilary organs of larva separated by a conspicuous groove; mandibular hook usually with five accessory teeth, rarely six; vestiture of PRP undescribed … *E*. funeralis*

### *Merodon* species

1a. PRP without spiracular setae. Anal segment without lappets (South African species) … *M*. bombiformis*

1b. PRP with spiracular setae. Anal segment with or without lappets (Palaearctic species) … 2

2a. ARP with 3–5 spiracular openings … 3

2b. ARP with two spiracular openings … 4

3a. Integument lacking setae (larvae and adults consistently associated to bulbs and flowers of *Drimia maritima* respectively) … ***M*. *luteihumerus***

3b. Integument covered with minute spinules (larvae associated to a wide range of commercial bulbs) … ***M*. *equestris***

4a. Mandibular hooks with accessory teeth. Two pairs of lappets … *M*. avidus*

4b. Mandibular hooks without accessory teeth. Three pairs of lappets, middle one consisting of two projections … 5

5a. Mandibular hooks not heavily sclerotised centrally. Lateral surface of PRP lacking obvious sculpturing … ***M*. *hurkmansi***

5b. Mandibular hooks heavily sclerotised all over (Fig 1D). Lateral surface of PRP conspicuously ornamented with points, short lines and domes (Fig 6D) … ***M*. *geniculatus***

### A compilation on the data available on early stages and host plants of the world *Eumerus* and *Merodon*

All data available in the literature on the early stages and host plants of *Eumerus* and *Merodon* are compiled and presented in Table 1. Speight et al [53] reinstated *Eumerus funeralis* Meigen, 1822 as the correct name for *Eumerus tuberculatus* Rondani, 1857 of authors, including Hodson [54, 55, 56]. However, we provide separate entries for these two names in Table 1 because most host plant and early stage data on this species appear in literature under the name *E*. *tuberculatus*.

## Discussion

Morphologically, all four species of Syrphidae studied in the present paper possess well-sclerotised mandibular hooks of different sizes, those of *Merodon* being larger than those of *Eumerus*, and, within *Eumerus*, those of *E*. *nudus* being the largest (Fig 1). In addition, all three studied *Eumerus* species have accessory teeth that surely assist the mouth hooks in rasping and scrapping solid tissue. Nevertheless, head skeletons of all these and other *Eumerus* species also have pharyngeal ridges [31]. Such structures indicate the ability of these species to feed on the fluids, and most probably the fungi and bacteria, associated with decay, as suggested by other studies focused on saprophagous hoverflies dependent on decomposing plant material [99, 100]. *Eumerus* larvae develop better in previously decayed material, suggesting their more saprophagous than phytophagous feeding regime [65, 101]. However, the larva of at least *E*. *nudus* appears to be capable of generating decay in intact plant tissue by mechanically damaging it and increasing the surface area to be attacked by microorganisms causing decay. Similarly, the larvae of *E*. *compertus* and *E*. *tricolor* have large mandibular hooks for feeding on intact plant tissue of *Cistanche* sp and *Tragopogon* spp plants, respectively (see Table 1). This is also important information that must be considered when searching for early stages of *Eumerus* in the field, as *Eumerus* larvae could be infesting a wider range of habitats than *Merodon*, both intact and liquefied plant tissues. In contrast, *M*. *geniculatus* lacks mandibular lobes but has the large heavily sclerotised mandibular hooks of other known *Merodon* larvae, suggesting a strict diet of living plant tissue, ripping apart the flesh of the bulbs where it lives.

Differences between the PRP lengths of *Eumerus* and *Merodon*, which is shorter in *Merodon* than in *Eumerus*, show that *Eumerus* is able to access air pockets within more liquefied materials while *Merodon* prevents its PRP being blocked by the decaying material left behind its larva in the excavated tunnels [47]. However, *E*. *compertus* and *E*. *tricolor* also have short PRP [59, 86], probably adapted to live in the tunnels and cavities they produce in their host plants. Special attention must be focused on the PRP of *E*. *hungaricus*, which is asymmetric in all three studied specimens (Fig 5A), a remarkable feature not seen before in other known *Eumerus* species. Additionally, none of our four species, neither *Eumerus* nor *Merodon*, have mesothoracic prolegs. The similar *E*. *obliquus* and *E*. *etnensis* are the only described species which have mesothoracic prolegs [4, 38].

Sampling methods for early stages of hoverflies living in underground storage organs of plants remain simple, and there is a need for innovation and a lot more effort in fieldwork. According to the current information on associations between *Eumerus* and *Merodon* and their host plants, their preference for geophytes makes the search for their host plants very complicated when plants are in their dormant state; even when the plant is visible, they do not always have symptoms of the presence of larvae inside their storage organs. It is very important to know the host plant of the hoverfly species being sought, as well as the development time in order to save time when digging for immature stages of hoverflies inside underground storage organs. This information about the host plants may be inferred by field observation of hoverfly behaviour during oviposition on plants. The knowledge on reproductive behaviour of both genera is still imprecise and biased. So far the majority of early stages found by different authors have been a result of extensive searches in similar plant biotypes. Our experience during field work tells us that an approach using adult behaviour in the wild, prior to the collection of early stages, helps greatly in finding early stages, although there is an important chance factor.

Other species of *Eumerus*, including *E*. *nudus*, have also been recently found in *A*. *ramosus* [12]. Plant species identification is important when studying insect-plant associations. *Asphodelus cerasiferus* distribution expands to the North of Spain whereas *A*. *ramosus* distribution is limited to the South and East of Spain. The taxonomic concept of *A*. *cerasiferus* includes some descriptions of *A*. *ramosus non Linnaeus* [102], so that those findings of the host plant of *E*. *nudus* of Speight and Garrigue from the western French Pyrenees [12] seem to belong to *A*. *cerasiferus* rather than *A*. *ramosus*

*E*. *hungaricus* puparia were obtained in 2010 from wild bulbs of *N*. *confusus* (*Amayllidaceae*) in Sierra de Béjar (1000 m), in the mountain area of Salamanca province, situated in Central-Western Spain. *E*. *strigatus* is known to be a pest of different cultivated plants of commercial interest (see Table 1). *M*. *geniculatus* larvae were obtained from different species of *Narcissus* in 2010. From the Natural Park of Sierra de Mariola, Alicante (SE Spain), specimens of *M*. *geniculatus* were collected, as described above, from wild bulbs of *N*. *dubius*. However, from the Botanical Garden of Torretes (Ibi, Alicante, SE Spain), *M*. *geniculatus* specimens were taken from cultivated bulbs of *Narcissus triandrus* subsp. *pallidulus* (Graells) Rivas Goday, *Narcissus rupicula* Dufour and *Narcissus tazetta* L. As the specimens from the Botanical Garden of Torretes were taken from commercially obtained or exchanged bulbs from other botanical gardens, the relationships of this *M*. *geniculatus* with the bulbs where it was found is in doubt; specimens could have accidentally come inside previously bought infested bulbs or could have been infected right at the Botanical Garden of Torretes, according to the *M*. *geniculatus* distribution [32]. Another species of *Merodon*, *M*. *equestris*, is widely known for being a horticultural pest and *M*. *geniculatus* might behave as a pest too. In any case, the genus *Merodon* tends to be widely associated with *Narcissus* spp. along with other bulb plants containing toxic compounds as, for example, in the *D*. *maritima* fed on by *M*. *luteihumerus* [4]. Many more studies on larval biology, co-evolution or even about their ability to digest or eliminate the phytotoxins of these plant families are needed to elucidate the nutritional links between these hoverflies and their food plants.

All the information collated here starts to show the wide diversity of habitats and relationships *Eumerus* and *Merodon* establish with many different plants. Larvae of both *Eumerus* and *Merodon* seem to prefer underground storage organs of the families *Xhantorrhoeaceae* and *Hyacinthaceae*. Despite underground storage organs from monocot geophytes of these plant families being the main habitat for the early stages of both genera (Table 1), it is clear that species of *Eumerus* feed and live in both monocots and dicots, even in very different plants such as *Orobancheaceae*, *Cactaceae*, *Euphorbiaceae* or *Asteraceae*, but *Merodon* seems to only use monocot habitats. Although some *Eumerus* species appear to produce decay themselves in healthy parts of plants (e.g. *E*. *nudus*), the feeding regime of *Eumerus* larvae still remains clearly more saprophagous than phytophagous due to their morpho-functional adaptations and reported breeding sites, while *Merodon* is strictly phytophagous.

## References

[1] Myers N, Mittermeier RA, Mittermeier CG, da Fonseca GAB, Kent J. Biodiversity hotspots for conservation priorities. Nature. 2000;403: 853–858. doi: 10.1038/35002501 10706275 10.1038/35002501

[2] Farràs A. Les liliates o angiosperms monocotiledònies In: Masalles RM, Carreras-i-Raurell J, Farràs A, Ninot JM, editors. Història Natural dels Països Catalans, Plantes Superiors, volum 6 Barcelona: Enciclopèdia Catalana S.A; 1988 pp. 317–376.

[3] Blondel J, Aronson J. Biology and wildlife of the Mediterranean region. Oxford: Oxford University Press; 1999.

[4] Ricarte A, Marcos-García MA, Rotheray GE. The early stages and life histories of three *Eumerus* and two *Merodon* species (Diptera: Syrphidae) from the Mediterranean region. Entomol Fenn. 2008;19: 129–141.

[5] USDA. Phytosanitary Certificate Issuance & Tracking System (PCIT) Phytosanitary Export Database (PExD). https://pcit.aphis.usda.gov/pcit/. Cited 8 June 2016.

[6] Speight MCD. Species accounts of European Syrphidae 2016 Syrph the Net, the database of European Syrphidae (Diptera), vol. 93 Dublin: Syrph the Net publications; 2016.

[7] Free JB. Insect Pollination of Crops. London: Academic Press; 1993.

[8] Proctor M, Yeo P, Lack A. The Natural History of Pollination, The New Naturalist Series. New York: Harper & Collins Publishers; 1996.

[9] Ricarte A. Biodiversidad de sírfidos (Diptera: Syrphidae) y conservación de los hábitats en el Parque Nacional de Cabañeros, España. PhD Thesis, Universidad de Alicante. 2008. http://rua.ua.es/dspace/handle/10045/9663.

[10] Peterson A, Bartish IV, Peterson J. Effects of population size on genetic diversity, fitness and pollinator community composition in fragmented populations of *Anthericum liliago* L. Plant Ecol. 2008;198: 101–110.

[11] Andrić A, Šikoparija B, Obreht D, Đan M, Preradović J, Radenković S, et al DNA barcoding applied: identifying the larva of *Merodon avidus* (Diptera: Syrphidae). Acta Entomol Mus Natl Pragae. 2014;54(2): 741–757.

[12] Speight MCD, Garrigue J. Rearing *Eumerus nudus*, *E*. *olivaceus* and *E*. *pulchellus* (Diptera, Syrphidae) from asphodel, with notes on separation of *E*. *nudus and E*. *olivaceus*. Dipt Digest. 2014;21: 59–72.

[13] Thompson FC, Vockeroth JR. Family Syrphidae In: Evenhuis N, editor. Catalog of the Diptera of the Australasian and Oceanian regions. Honolulu: Bishop Museum Press; 1989 pp. 437–458.

[14] Johnson CW. Some additions to the dipteran fauna of New England. Psyche. 1910;17: 228–235.

[15] Gibson A. The occurrence of *Eumerus strigatus* FLN in Canada. Can Entomol. 1917;49(6): 190–191.

[16] Jones CR. New species of Colorado Syrphidae. Ann Entomol Soc Am. 1917;10: 219–231.

[17] Mackie DB. Note on the lesser Bulb or lunate fly. Monthly Bul Calif Dept Agr. 1922;11(10): 759.

[18] Weiss HB, Nicolay AS. *Eumerus strigatus* Fall., the lunate onion fly, in New Jersey. Entomol News. 1919;30: 27.

[19] Smith LM. Distinction between three species of *Eumerus* (Syrphidae, Diptera), with description of a new species. Pan-Pac Entomol. 1928;4: 137–139.

[20] Gerding MP, Cisternas EA, Aguilera AP, Apablaza JH. *Eumerus strigatus* (Fallen) (Diptera: Syrphidae) infestando Alliaceae en Chile. Agric Téc. 1999;59(2): 133–135.

[21] Marinoni L, Morales MN. The Second Record of the Genus *Eumerus* Meigen, 1822 (Diptera: Syrphidae) for the Neotropical Region and the First for Brazil. Proc Entomol Soc Wash. 2007;109(2): 493–495.

[22] Speight MCD, Hauser M, Withers P. *Eumerus narcissi* Smith (Diptera, Syrphidae), presence in Europe confirmed, with a redescription of the species. Dipt Digest. 2013;20: 17–32.

[23] Peck LV. Syrphidae In: Soos A, Papp L, editors. Catalogue of Palaeartic Diptera. Budapest: Akad. Kiado; 1988 pp. 11–230.

[24] Kuznetzov SY. A new Palaearctic species and new female of the genus *Eumerus* Meigen (Diptera, Syrphidae). Dipterological Research. 1992;3(1–2): 33–40.

[25] Doczkal D. Description of two new species of the genus *Eumerus* Meigen (Diptera, Syrphidae) from Corsica. Volucella. 1996;2(1–2): 3–19.

[26] Barkalov AV, Gharaei B. Description of a new species of the genus *Eumerus* (Diptera, Syrphidae) from Iran. Volucella. 2004;7: 105–109.

[27] Grković A, Vujić A, Radenković S, Chroni A, Petanidou T. Diversity of the genus *Eumerus* Meigen (Diptera, Syrphidae) on the eastern Mediterranean islands with description of three new species. Ann Soc Entomol Fr. 2016;51(4): 361–373.

[28] Markov Z, Nedeljković Z, Ricarte A, Vujić A, Jovičić S, Józan Z, et al Bee (Hymenoptera: Apoidea) and hoverfly (Diptera: Syrphidae) pollinators in Pannonian habitats of Serbia, with a description of a new *Eumerus* Meigen species (Syrphidae). Zootaxa. 2016;4154(1): 27–50. doi: 10.11646/zootaxa.4154.1.2 27615823 10.11646/zootaxa.4154.1.2

[29] Ståhls G, Vujić A, Pérez-Bañón C, Radenković S, Rojo S, Petanidou T. COI barcodes for identification of *Merodon* hoverflies (Diptera, Syrphidae) of Lesvos Island, Greece. Mol Ecol Resour. 2009;9: 1431–1438. doi: 10.1111/j.1755-0998.2009.02592.x 21564929 10.1111/j.1755-0998.2009.02592.x

[30] Vujić A, Radenković S, Ståhls G, Ačanski J, Stefanović A, Veselić S, et al Systematics and taxonomy of the ruficornis group of genus *Merodon* Meigen (Diptera: Syrphidae). Syst Entomol. 2012;37(3): 578–602.

[31] Rotheray GE, Gilbert F. The Natural History of hoverflies. Ceredigion: Forrest Text; 2011.

[32] Marcos-García MA, Vujić A, Mengual X. Revision of Iberian species of the genus *Merodon* (Diptera: Syrphidae). Eur J Entomol. 2007;104: 531–572.

[33] Vujić A, Marcos-García MA, Sarıbıyık S, Ricarte A. New data on the *Merodon* Meigen 1803 fauna (Diptera: Syrphidae) of Turkey including description of a new species and changes in the nomenclatural status of several taxa. Ann Soc Entomol Fr. 2011;47(1–2): 78–88.

[34] Vujić A, Radenković S, Ačanski J, Grković A, Taylor M, Şenol SG, et al Revision of the species of the *Merodon nanus* group (Diptera: Syrphidae) including three new species. Zootaxa. 2015;4006(3): 439–62. doi: 10.11646/zootaxa.4006.3.2 26623777 10.11646/zootaxa.4006.3.2

[35] Micó E, Marcos-García MA, Galante E. *Los insectos saproxílicos del Parque Nacional de Cabañeros*. Madrid: Organismo Autónomo de Parques Nacionales, Ministerio de Agricultura, Alimentación y Medio Ambiente; 2013.

[36] Jack JG. An enemy of Narcissus and Amaryllis. Garden and Forest. 1897;478: 154–156.

[37] Doucette CF, Latta R, Martin CH, Schopp R, Eide PM. Biology of the Narcissus Bulb Fly in the Pacific Northwest. Tech Bull U S Dep Agric. 1942;809: 1–63.

[38] Pérez-Bañón C, Marcos-García MA. Life history and description of the immature stages of *Eumerus pupariae* (Diptera: Syrphidae) developing in *Opuntia maxima*. Eur J Entomol. 1998;95: 273–380.

[39] Assem MA, Abdel AM, Yousef KEH. Efficiency of insecticidal dusts in controlling the bulb fly, *Eumerus amoenus* Loew on stored onion bulbs. Bull Entomol Soc Egypt. 1972;6: 217–219.

[40] Conijn CGM. Control of the large narcissus fly *Merodon equestris* Fab. (Diptera: Syrphidae) in the field. Meded Fac Landbouwwet, Rijksuniv Gent. 1990;55 (2b): 675–679.

[41] Stackelberg AA. Palaeartic species of the genus Eumerus Mg. (Diptera, Syrphidae). Tr Russ Entomol Obs (2000). 1961;48: 81–229.

[42] Vujić A, Šimić S. Genus *Eumerus* Meigen 1822 (Diptera: Syrphidae) in the area of the former Jugoslavia. Glasnik Prirod Muzeja u Beogradu B. 1999;49–50 (1995–1998): 173–190.

[43] Hartley JC. A taxonomic account of the larvae of some British Syrphidae. Proc Zool Soc London. 1961;136: 505–573.

[44] Rotheray GE. Colour Guide to Hoverfly larvae (Diptera: Syrphidae). Dipt Digest. 1993;9: 1–156.

[45] Hartley JC. The cephalopharyngeal apparatus of syrphid larvae and its relationship to other dipteral. Proc Zool Soc London. 1963;141: 261–280.

[46] Roberts MJ. The Structures of the Mouthparts of Syrphid Larvae (Diptera) in Relation to Feeding Habits. Acta Zool. 1969;51: 43–65.

[47] Rotheray GE, Gilbert F. Phylogeny of Palaeartic Syrphidae (Diptera): evidence from larval stages. Zool J Linn Soc. 1999;127: 1–112.

[48] Thompson FC. A key to the genera of the flower flies (Diptera: Syrphidae) of the Neotropical Region including descriptions of new genera and species and a glossary of taxonomic terms. Contrib Entomol Internat. 1999;3: 321–378.

[49] Szilády Z. Über Paläearktischen Syrphiden. IV. Ann Mus Nat Hung (Zool). 1940;33: 54–70.

[50] Doesburg PH. Redescription of *Eumerus hungaricus* Szilády. Entomol ber. 1960;20: 144–145.

[51] ICZN. International Code of Zoological Nomenclature. 4th ed London: The International Trust for Zoological Nomenclature; 1999.

[52] Séguy E. Dipteres Syrphides de l’Europe occidentale. Mem Mus Nat Hist Nat, Nouv Ser, A, Zoologie. 1961;23: 1–248.

[53] Speight MCD, Claussen C, Hurkmans W. Révision des syrphes de la faune de France: III—Liste alphabétique des espèces des genres *Cheilosia*, *Eumerus* et *Merodon* et Supplément (Diptera, Syrphidae). Bull Soc ent Fr. 1998;103: 403–414.

[54] Hodson WEH. The bionomics of the lesser bulb flies, *Eumerus strigatus*, Flyn., and *Eumerus tuberculatus*, Rond., in South-West England. Bull Entomol Res. 1927;23: 429–448.

[55] Hodson WEH. A comparison of the larvae of *Eumerus strigatus*, Flyn., and *Eumerus tuberculatus*, Rond. (Syrphidae). Bull Entomol Res. 1932;23: 247–249.

[56] Hodson WEH. A comparison of the immature stages of *Eumerus tuberculatus* Rond. and *Syritta pipiens* Lin. (Syrphidae). Bull Entomol Res. 1931;22: 55–58.

[57] Efflatoun HC. A monograph of Egyptian Diptera, Pt. 1: Syrphidae. Mem Egypt Entomol Soc. 1922;2(1): 1–123.

[58] Assem MA, Nasr ESA. A syrphid fly, *Eumerus amoenus* Loew injurious to onion in U.A.R. (Diptera: Syrphidae). Agric Res Rev (Cairo). 1967;45(2): 27–32.

[59] Waitzbauer W. *Eumerus compertus* Villeneuve (Dipt., Syrphidae); larve and puparium. Zool Anz. 1976;196: 16–22.

[60] Stuke JH. Phylogenetische Rekonstruktion der Verwandschaftsbeziehungen innerhalb der Gattung *Cheilosia* Meigen, 1822 anhand der Larvenstadien (Diptera: Syrphidae). Stud Dipterol (Supplement). 2000;8: 1–118.

[61] Hardy DE. Diptera: Brachycera II—Cyclorrhapha I (Vol. 11) In: Zimmerman E, editor. Insects of Hawaii. Honolulu: University of Hawaii Press; 1964 pp. 1–458.

[62] Miyasaka SC, Ogoshi RM, Tsuji GY, Kodani LS. Site and Planting Date Effects on Taro Growth: Comparison with Aroid Model Predictions. Agron J. 2003;95: 545–557.

[63] Báez M. Los Sírfidos de las Islas Canarias Monografías Sección IV (XV). Santa Cruz de Tenerife: Instituto de Estudios Canarios (Universidad de la Laguna); 1977.

[64] Latta R, Cole FR. A comparative study of the species of *Eumerus* known as the lesser bulb flies. Monthly Bul Calif Dept Agr. 1933;22: 142–152.

[65] Blanton FS, Spruijt FJ. The species of *Eumerus* on Long Island. J Econ Entomol. 1933;26: 514–515.

[66] Blanton FS. Some Dipterous Insects Reared from *Narcissus* Bulbs. J Econ Entomol. 1938;31(1): 113–116.

[67] De Moor FC. Notes on a syrphid fly, *Eumerus obliquus* (Fabricius) (Diptera: Syrphidae). Arnoldia. 1973;6(15): 1–7.

[68] Johansson N. Återfynd av rödhornig månblomfluga *Eumerus ruficornis* Meigen, 1822 (Diptera, Syrphidae) med noteringar kring artens ekologi. Entomol Tidskr. 2011;132: 5–10.

[69] Munk T. Svirrefluen *Eumerus sabulonum* (Fallén, 1817) (Syrphidae, Diptera) yngler i blamunke (*Jasione montana* L.). Flora og Fauna. 2000;106: 19–22.

[70] Brunel E, Cadou D. Syrphid larvae (Diptera: Syrphidae) mining the roots of artichoke (*Cynara scolymus*, L.) in Brittany. Dipt Digest. 1994;1: 69–71.

[71] Neboiss A. Comparative study of Victorian bulb flies, *Eumerus* species (Syrphidae, Diptera). Victorian nat. 1957;74: 3–11.

[72] Dufour ML. Histoire des metamorphoses de l’*Eumerus aeneus*, Macq. Mem Soc Sci Agric Lille. 1845; 197–200.

[73] Broadbent BM. Notes on the life history of the lesser bulb fly *Eumerus strigatus* Fallen. J Econ Entomol. 1925;18(1): 141–143.

[74] Wilcox J. The Lesser Bulb Fly, *Eumerus strigatus* Fallen, in Oregon. J Econ Entomol. 1926;19: 762–772.

[75] Keifer HH. Notes on Lesser Bulb Flies in California. Monthly Bul Calif Dept Agr. 1930;19(11): 760.

[76] Gyulai P. *Eumerus strigatus* Meig., pest of carrots. Novenyvedelem. 1980;16: 58–61.

[77] Collin JE. *Eumerus strigatus* Fallen and tuberculatus Rondani (Diptera, Syrphidae). Ent mon Mag. 1920;56: 102–106.

[78] Bean JL. *Eumerus strigatus* reared from potatoes. J Econ Entomol. 1947;40: 452–454. 10.1093/jee/40.3.452a 20344770

[79] Doane JF. Attraction of the lesser bulb fly *Eumerus strigatus* (Diptera: Syrphidae) to decomposing oatmeal. N Z Entomol. 1983;7(4): 419.

[80] Kizil S, Arslan N, Ölmez-Bayhan S, Khawar KM. Effects of different planting dates on improving yield of *Fritillaria imperialis* L. and *Fritillaria persica* L. bulbs damaged by small narcissus fly (*Eumerus strigatus* Fallen). Afr J Biotechnol. 2008;7(24): 4454–4458.

[81] Heiss EM. A classification of the larvae and puparia of the Syrphidae of Illinois exclusive of Aquatic Forms. III Biol Monogr. 1938;16(4): 142, 17 pls.

[82] Kanervo V. Die Unterscheidung der Larven und Puppen von *Eumerus tuberculatus* Rond. und *E*. *strigatus* Fall. Ann Ent Fenn. 1942;8: 227–233.

[83] Dixon TJ. Key to and descriptions of the third instar larvae of some species of Syrphidae (Diptera) occurring in Britain. T Roy Ent Soc London. 1960;112(13): 345–379.

[84] Dušek J, Láska P. Prispevek k poznani larev pestrenek (Syrphidae, Diptera) II. Acta rerum nat dist Silesiae. 1960;21: 299–320.

[85] Dušek J, Láska P. Prispevek k poznani larev pestrenek (Syrphidae, Diptera) III. Acta rerum nat dist Silesiae. 1961;22: 513–540.

[86] Arzone A. Reperti biologici su *Eumerus tricolor* Meigen, nocivo alle coltivazioni di *Tragopogon porrifolius* L. in Piemonte (Dipt. Syrphidae). Ann Fac Sci Agr Univ Torino. 1971–1972;7: 17–52.

[87] Arzone A. *Tragopogon pratensis* L., ospite natural di *Eumerus tricolor* Meigen (Dipt. Syrphidae). Ann Fac Sci Agr Univ Torino. 1973;8: 55–66.

[88] Martin CH. Notes on the larval feeding habits and life history of *Eumerus tuberculatus* Rondani. Bull Brooklyn Ent Soc. 1934;29: 27–36.

[89] Popov GV. *Merodon alexandri* spec. nov.–a new species of hoverfly (Diptera: Syrphidae) from the northern Black Sea Region. Stud Dipterol. 2010;16: 133–151.

[90] Reemer M, Goudsmits K. Oviposition observed in *Chrysotoxum cautum*, *C*. *vernal* and *Merodon avidus* (Diptera: Syrphidae). Volucella. 2004;7: 217–218.

[91] Stuckenberg BR. The immature stages of *Merodon bombiformis* Hull, a potential pest of bulbs in South Africa. J Entomol Soc South Afr. 1956;19(2): 219–224.

[92] Pehlivan E, Akbulut N. Some investigations on the syrphid species attacking on Narcissus in Karaburun (Izmir) and the biology and control measures of *Merodon eques* (F.) (Diptera). Tr J of Agriculture and Forestry. 1991;15: 47–81.

[93] Childs L. The large narcissus bulb fly. The monthly Bulletin of state Commission of Horticulture. 1914; 3: 73–76.

[94] Hodson WEH. The large narcissus fly, *Merodon equestris*, Fab. (Syrphidae). Bull Entomol Res. 1932;17: 373–385.

[95] Wallace B, Wallace ID. An unexpected rearing record for *Merodon equestris*. Hoverfly Newsletter. 1990;11: 8.

[96] Wilcox J, Mote DC. Observations on the life history, habits and control of the Narcissus Bulb Fly, *Merodan equestris* Fab. in Oregon. J Econ Entomol. 1927;20: 708–714.

[97] Hurkmans W. Ethology and ecology of *Merodon* in Turkey (Diptera: Syrphidae). Entomolog Ber. 1988;48(7): 107–114.

[98] Stepanenko OV, Popov GV. On the immature stages biology of Merodon nigritarsis Rondani, 1845 (Diptera: Syrphidae). The Karkov Ent Soc Gazette. 1997;5(2): 40–43.

[99] Martínez-Falcón AP, Durbán A, Latorre A, Antón J, Marcos-García MA. Bacteria Associated with Copestylum (Diptera, Syrphidae) Larvae and Their Cactus Host *Isolatocereus dumortieri*. PLoS ONE. 2011;6(11): e27443 doi: 10.1371/journal.pone.0027443 22132101 10.1371/journal.pone.0027443 PMC3223168

[100] Sánchez-Galván IR, Ferrer J, Galante E, Marcos-García MA. Bacteria and Hoverflies (Diptera: Syrphidae) in Tree Hollows From the Iberian Mediterranean Forest. Environ Entomol. 2016;46(1): 137–142. 10.1093/ee/nvw158 28025224

[101] Creager DB, Spruijt FJ. The relation of certain fungi to larval development of *Eumerus tuberculatus*. Ann Entomol Soc Am. 1935;28: 425–437.

[102] Díaz-Lifante Z. *Asphodelus* L In: Castroviejo S, Aedo C, Laínz M, Muñoz-Garmendia F, Nieto-Feliner G, Paiva J, et al, editors. Flora Ibérica. Madrid: Real Jardín Botánico, CSIC; 2013;20 pp. 276–308.

